# Impact of gestational diabetes mellitus on neonatal outcomes in small for gestational age infants: a multicenter retrospective study

**DOI:** 10.1007/s00404-024-07587-y

**Published:** 2024-06-14

**Authors:** Ayala Hirsch, Tzuria Peled, Shaked Schlesinger, Hen Y. Sela, Sorina Grisaru-Granovsky, Misgav Rottenstreich

**Affiliations:** 1 Department of Obstetrics and Gynecology, Shaare Zedek Medical Center, Affiliated with the Hebrew University School of Medicine, 12 Bayit Street, 91031 Jerusalem, Israel; 2 Department of Military Medicine and “Tzameret”, Faculty of Medicine, Israel Defense Forces, Hebrew University of Jerusalem, and Medical Corps, Jerusalem, Israel; 3 Department of Nursing, Jerusalem College of Technology, Jerusalem, Israel

**Keywords:** Gestational diabetes mellitus, Small for gestational age, Intrauterine growth restriction, Fetal growth restriction, Birth weight, Perinatal outcomes, Neonatal outcomes, Hypoglycemia

## Abstract

**Objective:**

To evaluate obstetric and perinatal outcomes among small for gestational age (SGA) infants born to patients diagnosed with Gestational diabetes mellitus (GDM).

**Materials and methods:**

A multicenter retrospective cohort study between 2005 and 2021. The perinatal outcomes of SGA infants born to patients with singleton pregnancy and GDM were compared to SGA infants born to patients without GDM. The primary outcome was a composite adverse neonatal outcome. Infants with known structural/genetic abnormalities or infections were excluded. A univariate analysis was conducted followed by a multivariate analysis (adjusted odds ratio [95% confidence interval]).

**Results:**

During the study period, 11,662 patients with SGA infants met the inclusion and exclusion criteria. Of these, 417 (3.6%) SGA infants were born to patients with GDM, while 11,245 (96.4%) were born to patients without GDM. Overall, the composite adverse neonatal outcome was worse in the GDM group (53.7% vs 17.4%, p < 0.01). Specifically, adverse neonatal outcomes such as a 5 min Apgar score < 7, meconium aspiration, seizures, and hypoglycemia were independently associated with GDM among SGA infants. In addition, patients with GDM and SGA infants had higher rates of overall and spontaneous preterm birth, unplanned cesarean, and postpartum hemorrhage. In a multivariate logistic regression assessing the association between GDM and neonatal outcomes, GDM was found to be independently associated with the composite adverse neonatal outcome (aOR 4.26 [3.43–5.3]), 5 min Apgar score < 7 (aOR 2 [1.16–3.47]), meconium aspiration (aOR 4.62 [1.76–12.13]), seizures (aOR 2.85 [1.51–5.37]) and hypoglycemia (aOR 16.16 [12.79–20.41]).

**Conclusions:**

Our study demonstrates that GDM is an independent risk factor for adverse neonatal outcomes among SGA infants. This finding underscores the imperative for tailored monitoring and management strategies in those pregnancies.

## Introduction

Gestational diabetes mellitus (GDM), defined as glucose intolerance first identified during pregnancy [1], affects 14% of pregnancies and is rapidly escalating in prevalence globally [2]. Typically, the hyperglycemic state associated with GDM results in fetal hyperinsulinemia, leading to the development of large for gestational age (LGA) infants with specific associated morbidity [3, 4]. Surprisingly, up to 7% of infants born to patients with GDM exhibit the opposite outcome—being small for gestational age (SGA) [3]. SGA infants, defined as those with a birth weight below the 10th percentile for their gestational age [5] often face a heightened risk of adverse perinatal outcomes [6]. Notably, infants born to patients with GDM or infants that are SGA share similar risks for long-term complications such as obesity, type 2 diabetes, hypertension, and ischemic heart disease, hinting at a potential shared biological pathway between these two conditions [7–9].

Despite these insights, the existing literature on the intersection between GDM and SGA infants remains sparse [8, 10].

This study aims to explore the obstetric and perinatal outcomes of SGA infants born to patients with GDM compared to those without.

## Materials and methods

A multicenter retrospective cohort study was conducted from 2005 to 2021, utilizing computerized medical records from two university-affiliated medical centers in Jerusalem, Israel: shaare zedek medical center (SZMC) and bikur holim medical center (BHMC). These medical centers collectively account for approximately 16% of all deliveries in Israel, with an average annual volume of 20,000 deliveries.

For the study’s purpose, patients with singleton pregnancies diagnosed with SGA infants were enrolled. Exclusion criteria encompassed patients with unknown glycemic status due to a lack of diagnostic tests, pre-pregnancy diabetes mellitus, multifetal gestation, pre-viable deliveries before 24 + 0 weeks, pre-delivery fetal death, and with known major malformations, genetic disorders, or infection.

GDM was defined either by an appropriate diagnosis in the patient’s medical record or an abnormal result in glucose tolerance test using the “two-step” method; In Israel, all pregnant women receive antenatal care coverage under the National Health Plan and are routinely screened for GDM via a 50 g oral GCT between 24 and 28 gestational weeks. Women with a plasma glucose concentration of ≥ 140 mg/dL at the GCT or with other risk factors for GDM and a result of ≥ 130 mg/dL are referred to OGTT. A diagnosis of GDM is made if the woman meets specific criteria, which include fasting 3 h 100 g OGTT values (two or more of the following values: fasting ≥ 95 mg/dL, 1 h ≥ 180 mg/dL, 2 h ≥ 155 mg/dL, and/or 3 h ≥ 140 mg/dL [11]) or GCT test result ≥ 200 mg/dL [12].

SGA was defined by a birth weight below the 10th percentile for gestational age and gender based on the birth weight curves of the Israeli population [13].

Both medical centers follow similar departmental protocols regarding the antepartum and intrapartum management of SGA and GDM that align with the criteria defined in the guidelines of the Israeli Committee of Obstetrics and Gynecology [14, 15].

Demographic and obstetric characteristics of patients with small-for-gestational age infant with and without Gestational diabetes were evaluated including age, parity, pregnancy HTN disorders, hypertensive disorders of pregnancy and obesity.

‘Pregnancy HTN disorders’ was defined as all hypertensive disorders occurring in pregnant patients, including those with pre-existing chronic hypertension. ‘Hypertensive disorders of pregnancy’ refer specifically to pregnancy-induced hypertension disorders, such as gestational hypertension, preeclampsia, and HELLP syndrome, which manifest during pregnancy and are not present before gestation.

The primary outcome was defined as adverse neonatal outcome including at least one of the following: perinatal mortality, neonatal intensive care unit (NICU) admission, Apgar score less than 7 at 5 min, meconium aspiration, hypoglycemia, neonatal jaundice, convulsions, shoulder dystocia or clavicular fracture, need for mechanical ventilation, respiratory distress syndrome or transient tachypnea of the newborn (TTN), necrotizing enterocolitis, intracranial hemorrhage, or neonatal sepsis [10]. Hypoglycemia is defined in our institution as blood sugar levels below 50 mg/dL before feeding. Monitoring of blood sugar levels in neonates at high risk for hypoglycemia is conducted in accordance with the guidelines of the American Academy of Pediatrics [16]. Regular Monitoring for neonates born to diabetic mothers is conducted for 24 h postpartum while monitoring for small-for-gestational-age neonates is conducted for 12 h.

The secondary outcomes included the individual components of the composite outcome and the following obstetric and maternal outcomes; Gestational age at delivery, Preterm birth (PTB, < 37 + 0 weeks of gestation), postpartum hemorrhage (PPH, Estimated blood loss of over 1000 mL and/or hemoglobin drop > 3 gr/dL and/or transfusion of blood products), placental abruption, maternal intensive care unit (ICU) admission and cesarean delivery (CD) rate.

Both SZMC and BHMC maintain electronic medical record databases. Delivery data are updated in real-time by attending healthcare professionals and audited periodically by trained technical personnel to ensure data validity and eliminate information bias. For this study, we extracted relevant maternal and neonatal records from this database, and personal information for each patient was pseudonymized before analysis, with all identifiable data replaced by unique codes.

The study group consisted of patients with GDM and SGA infants and they were compared to those with SGA infants without GDM (control group).

The study was approved by the SZMC’s institutional ethics committee (IRB: 0253-23-SZMC) that oversees both medical center research activities, adhering to the declaration of Helsinki, and given its retrospective and unidentified nature, informed consent was waived.

### Statistical methods

Nominal variables were described using proportions and compared using the appropriate statistical tests, such as the chi-square test or Fisher’s exact test. Continuous variables, which did not follow a normal distribution, were presented as means ± standard deviation (SD) or medians with interquartile ranges (IQR). These continuous variables were analyzed using the unpaired student’s t-test or Mann–Whitney test, depending on the distribution. A p-value of less than 0.05 was considered statistically significant.

The association between GDM and neonatal outcomes was adjusted to maternal age, gravidity, parity, number of previous CDs, fertility Treatments, hypertensive disorders of pregnancy, and gestational age at delivery using a multivariate logistic regression model. Adjusted odds ratios (aOR) with 95% confidence intervals (CIs) were reported to measure the strength of the association. All statistical tests were two-sided. The statistical analyses were performed using SPSS software (version 25, IBM, Armonk, NY).

## Results

**Fig. 1:**
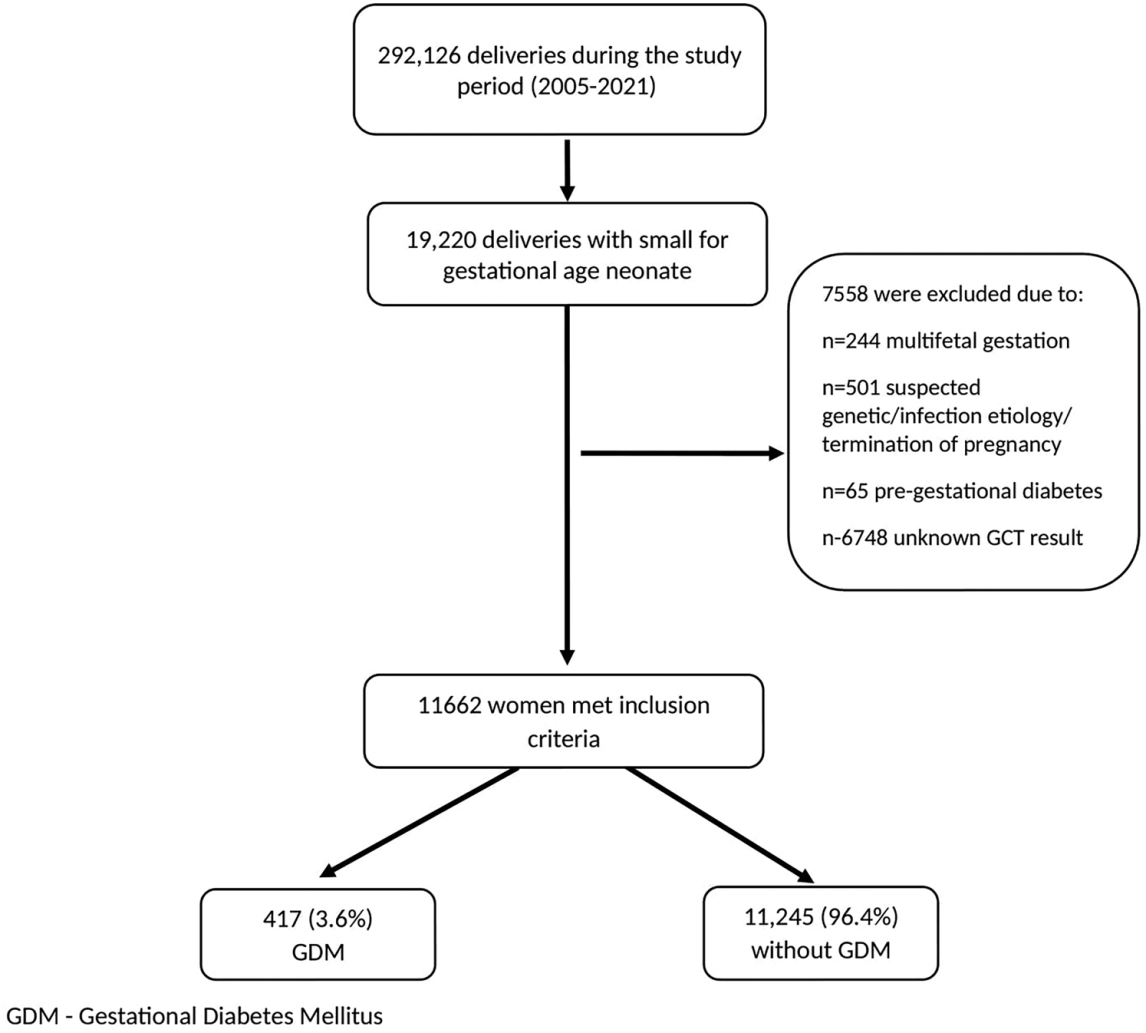
Study population schematic flowchart

During the study period, there were 292,126 deliveries in our hospitals. Of these, 19,220 (6.6%) involved SGA infants. After applying our inclusion criteria, 11,662 cases were included, with 417 (3.6%) diagnosed with GDM and 11,245 (96.4%) born to patients without GDM (Fig. 1).

In Table 1, we provide an overview of the general demographics and obstetric characteristics of the study population. Patients with GDM were of advanced maternal age, with higher parity and higher rates of obesity, smoking, hypertensive disorders (both chronic and pregnancy-induced), fertility treatment, and previous CDs.

**Table 1:** Demographic and obstetric characteristics of women with small-for-gestational age infant with and without Gestational diabetes

	Normal glycemic status n = 11,245	Gestational diabetes n = 417	p value
Maternal age, years	27.4 ± 5.6	32.4 ± 6.4	< 0.01
Gravidity	2.9 ± 2.5	4.3 ± 3.5	< 0.01
Parity	2.5 ± 2	3.6 ± 2.9	< 0.01
Primipara	4916 (43.7%)	136 (32.6%)	< 0.01
Previous cesarean delivery, any	1181 (10.5%)	88 (21.1%)	< 0.01
Fertility treatments	494 (4.4%)	36 (8.6%)	< 0.01
Hypertensive disorders of pregnancy	544 (4.8%)	57 (13.7%)	< 0.01
Chronic HTN	63 (0.6%)	14 (3.4%)	< 0.01
Pregnancy HTN disorders	498 (4.4%)	45 (10.8%)	< 0.01
Smoking	360 (3.3%)	21 (5.4%)	0.02
Obesity (BMI > 30)	278 (8.8%)	31 (16.1%)	< 0.01

Data are mean ± standard deviation; number (%)

*HTN* hypertension; *BMI* body mass index

Table 2 presents the neonatal characteristics of both groups.

**Table 2:** Neonatal outcomes among the study groups

	Normal glycemic status n = 11,245	Gestational diabetes n = 417	p value
Primary outcome			
Composite adverse neonatal outcome*	1957 (17.4%)	224 (53.7%)	< 0.01
Secondary outcomes			
Birthweight, grams	2564.4 ± 315.6	2447.6 ± 358.7	< 0.01
Percentile < 1%ile	604 (5.4%)	20 (4.8%)	0.61
Percentile < 5%ile	4175 (37.1%)	168 (40.3%)	0.19
Percentile 5–10%ile	6470 (57.5%)	229 (54.9%)	0.29
5 min Apgar score < 7	164 (1.5%)	16 (3.9%)	< 0.01
NICU admission	935 (8.3%)	63 (15.1%)	< 0.01
Meconium aspiration syndrome	28 (0.2%)	6 (1.4%)	< 0.01
Jaundice	807 (7.2%)	32 (7.7%)	0.70
TTN	207 (1.8%)	17 (4.1%)	< 0.01
Mechanical ventilation	159 (1.4%)	17 (4.1%)	< 0.01
Seizures	113 (1%)	12 (2.9%)	< 0.01
Erb’s palsy/fracture of clavicle	8 (0.1%)	0 (0%)	0.59
Hypoglycemia	406 (3.6%)	177 (42.4%)	< 0.01
Sepsis	45 (0.4%)	0 (0%)	0.20
Encephalopathy	8 (0.1%)	1 (0.2%)	0.22
Intracranial hemorrhage	30 (0.3%)	2 (0.5%)	0.41
Birth asphyxia	106 (0.9%)	5 (1.2%)	0.60

Data are mean ± standard deviation; number (%)

*NICU* neonatal intensive-care unit, *TTN* transient tachypnea of the newborn

*Including at least one of outcomes: NICU admission, meconium aspiration syndrome, jaundice, TTN, mechanical ventilation, seizures, Erb’s palsy/fracture of clavicle, hypoglycemia, sepsis, encephalopathy, intracranial hemorrhage and birth asphyxia

### Primary outcome

The GDM group exhibited worse rates of composite adverse neonatal outcomes (53.7% vs. 17.4%, p < 0.01).

### Secondary outcomes

SGA infants born to patients with GDM had lower birth weights (2447.6 vs. 2564.4, p < 0.01) without a difference in weight percentile groups. The GDM group had a significantly higher rate of NICU admission (15.1% vs. 8.3%, p < 0.01), lower 5 min Apgar scores < 7 (3.9% vs. 1.5%, p < 0.01), a higher rate of meconium aspiration (1.4% vs. 0.2%, p < 0.01), and higher rates of TTN, mechanical ventilation, seizures, and hypoglycemia (4.1% vs. 1.8%, 4.1% vs. 1.4%, 2.9% vs. 1%, and 42.4% vs. 3.6%, respectively, p < 0.01). However, other neonatal characteristics, including birth trauma, sepsis, and jaundice, did not differ between the groups.

Table 3 displays the delivery and obstetric outcomes. The GDM group had a higher rate of delivery induction (32.5% vs. 20.4%, p < 0.01), earlier gestational age at delivery (38.5 vs. 39.2 weeks, p < 0.01), a higher rate of PTB < 37 weeks (4.5% vs. 7.9%, p < 0.01), and spontaneous PTB (3.6% vs. 1.7%, p < 0.01). The GDM group showed a significantly higher rate of PPH (11.8% vs. 8.6%, p < 0.03), with no difference in blood product transfusion. Both planned and unplanned cesarean rates were significantly higher in the GDM group (15.8% vs. 8.9% and 19.9% vs. 7.3%, respectively, p < 0.01). Rates of maternal ICU admission and placental abruption did not differ between groups.

**Table 3:** Obstetric and maternal outcomes among the study groups

	Normal glycemic status n = 11,245	Gestational diabetes n = 417	p value
Induction of labor	2212 (20.4%)	122 (32.5%)	< 0.01
Oxytocin augmentation of labor	7225 (64.3%)	259 (62.1%)	0.37
Gestational age at delivery	39.2 ± 1.6	38.5 ± 1.8	< 0.01
PTB < 37 week	507 (4.5%)	33 (7.9%)	< 0.01
Spontaneous PTB < 37 week	191 (1.7%)	15 (3.6%)	< 0.01
Induced PTB < 37	316 (2.8%)	18 (4.3%)	0.07
PTB < 34 week	79 (0.7%)	8 (1.9%)	< 0.01
PTB < 32 week	34 (0.3%)	5 (1.2%)	< 0.01
PTB < 28 week	2 (0%)	0 (0%)	0.79
Maternal ICU admissions	5 (0%)	0 (0%)	0.67
Postpartum hemorrhage	971 (8.6%)	49 (11.8%)	0.03
Placental abruption	239 (2.1%)	7 (1.7%)	0.53
Blood products transfusion	114 (1%)	4 (1%)	0.91
Unplanned cesarean	996 (8.9%)	66 (15.8%)	< 0.01
Planned cesarean	823 (7.3%)	83 (19.9%)	< 0.01
Caesarean, total	1782 (15.8%)	146 (35%)	< 0.01

Data are mean ± standard deviation; number (%)

*PTB* preterm birth; *ICU* intensive care unit

Table 4 presents the results of a multivariate logistic regression assessing the association between GDM and neonatal outcomes, after controlling for significant covariates. GDM was independently associated with the composite adverse neonatal outcome (aOR 4.26, 95%CI 3.43–5.3) as well as with 5 min Apgar score < 7 (aOR 2, 95%CI 1.16–3.47), meconium aspiration (aOR 4.62, 95%CI 1.76–12.13), seizures (aOR 2.85, 95%CI 1.51–5.37), and hypoglycemia (aOR 16.16, 95%CI 12.79–20.41). NICU admission, mechanical ventilation, and TTN were no longer significantly increased after adjustment.

**Table 4:** Crude rate, odds ratio (OR) adjusted OR and 95% confidence intervals (CI) of composite adverse neonatal outcome among small -for-gestational age neonates with and without gestational diabetes mellitus

Outcome	OR* (95% CI)	aOR** (95% CI)
Composite adverse neonatal outcome***	5.51 (4.52–6.72)	4.26 (3.43–5.3)
5 min Apgar score < 7	2.7 (1.6–4.56)	2 (1.16–3.47)
NICU admission	1.96 (1.49–2.59)	1.05 (0.75–1.47)
Meconium aspiration syndrome	5.85 (2.41–14.2)	4.62 (1.76–12.13)
TTN	2.27 (1.37–3.75)	1.62 (0.94–2.79)
Mechanical ventilation	2.96 (1.78–4.93)	1.75 (0.98–3.12)
Seizures	2.92 (1.6–5.34)	2.85 (1.51–5.37)
Hypoglycemia	19.69 (15.83–24.48)	16.16 (12.79–20.41)

*NICU* neonatal intensive-care unit, *TTN* transient tachypnea of the newborn.

*Normal glycemic status serves as the referent group.

**Adjusted for maternal age, years, gravidity, parity, number of previous CDs, fertility treatments, hypertensive disorders of pregnancy and gestational age at delivery.

***Including at least one of outcomes: NICU admission, meconium aspiration syndrome, jaundice, TTN, mechanical ventilation, seizures, Erb’s palsy/fracture of clavicle, hypoglycemia, sepsis, encephalopathy, intracranial hemorrhage and birth asphyxia

## Discussion

In this retrospective cohort study, we aimed to evaluate the outcomes of SGA infants born to patients with GDM. Our data revealed that the composite neonatal morbidity was significantly more prevalent among SGA infants born to patients with GDM compared to SGA infants born to patients with no GDM. Specifically, adverse neonatal outcomes such as hypoglycemia, seizures, 5 min Apgar score < 7, and meconium aspiration, were independently associated with GDM among SGA infants. In addition, patients with GDM and SGA infants had higher rates of PTB, PPH, and unplanned CD.

Hypoglycemia is a common metabolic disturbance within the initial hours of an infant's life due to the abrupt cessation of glucose infusion from the placenta, causing a decline in the infant’s blood glucose concentration [17]. For most healthy infants, this transitional phase of neonatal hypoglycemia is brief, transient, and asymptomatic.

However, SGA infants born to patients with GDM face a higher risk of experiencing more severe or prolonged hypoglycemia [18]. This heightened risk is attributed to factors such as insufficient glycogen or fat tissue reserves and increased glucose utilization due to excessive insulin production due to the intra-uterine diabetic environment [19, 20]. Consequently, when blood sugar levels drop too low, the brain doesn’t receive adequate glucose, potentially leading to various neurological symptoms, including a higher rate of seizures observed in these infants [21]. Additionally, The elevated rates of 5 min Apgar score < 7 and meconium aspiration may be associated with the effects of GDM on endothelial dysfunction and the exacerbation of chronic fetal hypoxia [7, 22].

Previous research has primarily focused on outcomes of SGA newborns born to patients with GDM, often comparing them with appropriate for gestational age (AGA) or LGA newborns born to patients with GDM. Garcia-Patterson et al. [3] noted that SGA newborns had significantly higher rates of low 1 min Apgar scores and hypoglycemia compared to their LGA and AGA counterparts, mirroring our observations of increased vulnerability in SGA newborns to such complications. Similarly, Esakoff’s study [23] highlighted an increased risk of respiratory distress syndrome (RDS), intrauterine fetal demise (IUFD), neonatal demise, hypoglycemia, and jaundice in term SGA newborns compared to AGA newborns born to patients with GDM. Furthermore, Barquiel et al. [24] reported an additive effect of SGA status on the occurrence of neonatal complications like hypoglycemia, hyperbilirubinemia, polycythemia, and perinatal death, alongside a higher prevalence of maternal hypertension and preeclampsia in the SGA group in compare to AGA and LGA groups. In a retrospective cohort study conducted by Wang et al. [25] aimed to discern the characteristics of stillbirths within the context of diabetic pregnancies, IUGR was identified in 16% of 37 diabetic patients who encountered stillbirth. Specifically, within the subset of patients diagnosed with GDM and stillbirth, IUGR was identified in 26% of cases, emerging as a prominent characteristic associated with stillbirth in this particular population. Our findings align with the study of Chen et al. [10] which compared SGA newborns born to patients with GDM to those born to patients without GDM and found an increased risk for adverse outcomes, including low Apgar scores, early thrombocytopenia, hypoxic-ischemic encephalopathy, hypoglycemia, and pulmonary hemorrhage among the GDM group. However, this study by Chen et al. included only term infants, thereby excluding severe cases of growth restrictions. In addition, this study included infants with diverse malformations. In contrast, our study intentionally omitted such cases to present a more comprehensive overview of the associated morbidity in this population. In terms of CD rates, our study identified that SGA newborns born to patients with GDM were more likely to be delivered via cesarean, both elective and emergency, diverging from Chen et al. [10], who reported a lower rate of CD among GDM-associated SGA deliveries. This variance may reflect different clinical management approaches or different background clinical characteristics between the studies as a higher prevalence of previous CD was specifically observed in our study population, suggesting a distinct clinical profile that may further predispose these patients to surgical delivery. Notably, our findings of a higher rate of unplanned CD in SGA newborns, particularly those born to patients with GDM, can be attributed to increased fetal distress in this subgroup, and align with previous comprehensive studies and systematic reviews [20, 26].

In addition to the short-term pregnancy outcomes discussed above, numerous studies have demonstrated that SGA and GDM separately pose long-term risks for adverse outcomes in infants [7, 9, 27]. A study by Neimark et al. demonstrated that SGA infants born to patients with GDM exhibited a higher incidence of cardiovascular hospitalizations during childhood compared to SGA children born to patients without GDM [28].

Several studies have sought to characterize the risk factors linked to SGA infants in patients being treated for GDM. Within this specific cohort of patients, elevated incidences of hypertensive disorders, lower body mass index (BMI), inadequate maternal weight gain during pregnancy [29, 30], higher levels of glycemic control [31], and a higher frequency of infants deemed high risk for SGA among patients with GDM [32]. This reinforces our assertion that this subgroup presents unique risk factors and associated complications, which necessitate consideration during pregnancy, delivery, and post-natal care. It is noteworthy that our study exhibited significantly reduced rates of SGA neonates compared to those commonly reported in literature [5]. The lower rates of SGA in our study may be attributed to the unique demographic characteristics of our population, including a high percentage of high-order deliveries. Previous studies in our population have shown that higher parity can influence birth weight and reduce the SGA rate [33–35].

Several hypotheses have been posited to elucidate the pathophysiology of GDM and its association with fetal growth restriction [22, 37]. GDM is known to induce endothelial dysfunction, promoting the secretion of vasoactive substances and oxidative stress, which can lead to vascular insufficiencies such as thrombosis, hypertension, and atherosclerosis [22, 38–41]. The placental microangiopathy, impairs the placenta's capacity to provide sufficient nutrients and oxygen to the fetus, alterations in lipid metabolism, essential amino acids, and elevated inflammatory markers. All these mechanisms ultimately contribute to fetal growth restriction. Moreover, the hyperglycemic intrauterine environment associated with GDM may adversely affect fetal development, potentially increasing long-term cardiovascular risks for the offspring [9, 27].

In our study, we demonstrated that patients with GDM and SGA newborns were more likely to exhibit higher rates of obesity and hypertensive disorders, including chronic hypertension. These conditions, along with GDM, might exacerbate placental dysfunction, leading to impaired fetal growth. The prevalence of obesity and hypertension in these patients suggests an underlying metabolic syndrome, representing an ongoing assault on the maternal vascular system. This connection provides a clinical basis to suggest that these patients are at a higher risk of developing type 2 diabetes in the future, as their inability to manage the “stress test” of pregnancy indicates an increased vulnerability to metabolic disorders [42].

To further support our findings and hypotheses, future research could incorporate Doppler studies to assess placental pathology, offering direct evidence of the vascular abnormalities associated with GDM and SGA outcomes. Investigating maternal blood markers such as placental growth factor (PlGF), soluble fms-like tyrosine kinase-1 (sFlt-1), and inflammatory cytokines could further elucidate the mechanisms at play [22]. These biomarkers might shed light on the impact of GDM on placental function and its contribution to the development of SGA, thereby providing potential avenues for therapeutic intervention and improving maternal–fetal health outcomes.

### Strength and limitation

The strengths of our study include its multicenter design and the utilization of electronic medical records with real-time validation, minimizing retrospective study biases. Additionally, the comprehensive uniform coverage of all expenses related to antenatal, delivery, and postnatal care by the national health insurance enhances the reliability of our findings.

However, the study is not devoid of limitations. Its retrospective design inherently carries limitations, including potential biases not accounted for in the study design. The specific demographic characteristics of the population, especially the preference for larger family sizes, may limit the generalizability of the findings to other populations. The omission of Doppler studies of SGA infants and their correlation to placental pathology leaves unanswered questions about the underlying vascular pathophysiology, highlighting the need for further investigation in this area.

Additionally, the absence of long-term outcome data on SGA infants born to patients with GDM, such as catch-up growth and cardiovascular disease incidence, indicates a gap that necessitates further research.

## Conclusions

In conclusion, our multicenter retrospective cohort study sheds light on the association between GDM and adverse outcomes in SGA infants. The increased rates of adverse neonatal outcomes underscore the need for heightened clinical attention and management in pregnancies complicated by both GDM and SGA, advocating for personalized and proactive care strategies to safeguard their health and development. Further research should delve into placental pathology using Doppler studies and explore maternal blood markers to elucidate the mechanisms linking GDM to SGA outcomes.

## Data Availability

Not applicable.
